# Early Ciliovitreal Block following Ab Externo XEN Gel Implantation

**DOI:** 10.1155/2023/9775780

**Published:** 2023-10-06

**Authors:** Jason Ning

**Affiliations:** Omni Eye Services, 485 US Rt. 1 South, Building A, Iselin, NJ 08830, USA

## Abstract

**Purpose:**

To report a case of ciliovitreal block following ab externo implantation of a XEN gel stent for glaucoma.

**Methods:**

Case report.

**Results:**

An 84-year-old African American male underwent ab externo implantation of a XEN gel stent for elevated intraocular pressure. Several days after the surgery, the patient developed severe eye pain and reduced vision. Examination revealed ciliovitreal block (also called aqueous misdirection or malignant glaucoma) which was unresponsive to medical and laser treatment. Pars plana vitrectomy with anterior and posterior synechialysis and anterior chamber reformation were performed. The ciliovitreal block was resolved, and intraocular pressure brought to acceptable levels, but vision in that eye remained poor, likely due to the period of elevated pressure prior to treatment.

**Conclusion:**

Ciliovitreal block is a rare postsurgical (typically) form of secondary angle closure characterized by narrow or flat central and peripheral anterior chamber, anterior displacement of the lens-iris diaphragm, anterior rotation of ciliary processes, and (usually) elevated intraocular pressure. To our knowledge, this is the first reported case of ciliovitreal block occurring after ab externo XEN implantation. Early diagnosis and treatment are essential for preventing vision loss.

## 1. Introduction

Ciliovitreal block is a rare type of secondary angle closure that typically occurs following surgery. Many other names exist for this condition, the most common being aqueous misdirection and malignant glaucoma [1]. Intraocular pressure (IOP) is typically elevated but can be normal if there is a functioning glaucoma surgery. Because of the potential for extremely high IOP, ciliovitreal block is a serious condition which can lead to irreversible vision loss [2]. The etiology for ciliovitreal block is not fully understood, but different theories point toward misdirection of aqueous into the vitreal cavity [3],⁠ choroidal expansion [4], and annular ciliochoroidal detachments [5]⁠ as being the root cause.

The most common theory is that aqueous is misdirected into the vitreous cavity instead of the anterior chamber. As aqueous in the anterior chamber drains normally, misdirected aqueous continues to fill the vitreous cavity and the lens-iris diaphragm displaces anteriorly. Eventually, this leads to angle closure and potential collapse of the AC [1].⁠ Another theory by Epstein et al. and Quigley et al. proposes that ciliovitreal block is caused by choroidal expansion which reduces the available volume for the vitreous, posterior chamber, and anterior chamber, thus leading to angle closure. Both of these theories agree that increased IOP can lead to reduced vitreous permeability which prevents fluid from migrating from the vitreous cavity into the anterior chamber for drainage [6, 7].⁠ One final theory by Liebmann et al. proposes that cases of ciliovitreal block may be caused by aqueous misdirection and/or annular ciliochoroidal detachments which are not easily observable clinically [5].⁠

Diagnosis of ciliovitreal block is typically based on clinical findings and confirmatory ultrasound B-scan and ultrasound biomicroscopy (UBM). B-scan is used to confirm the absence of any choroidal detachments while UBM can visualize anterior rotation of ciliary processes and detect ciliochoroidal detachments (if present) [5].⁠

Treatment for ciliovitreal block can be medical, laser, or surgical. One caveat is that if an LPI does not exist, one should be made [2].⁠ Medical treatments can be trialed for several days but have mediocre success rates, and relapses may occur as medications are withdrawn. Medications used include cycloplegics/mydriatics, IOP lowering medications (carbonic anhydrase inhibitors, beta blockers, hyperosmotics, and alpha agonists), and steroids [8].⁠ Laser treatment typically involves YAG posterior capsulotomy and anterior hyaloidotomy to attempt to make the eye unicameral and allow fluid flow from the vitreous cavity into the posterior-then-anterior chamber [9].⁠ Cyclophotocoagulation is sometimes used as well to reduce aqueous production and attempt to disrupt the anterior hyaloid [10].⁠

Surgical treatment is typically aimed at disrupting the anterior hyaloid to allow fluid flow into the anterior chamber for drainage. Another caveat is that if the eye is still phakic, phacoemulsification or lensectomy is recommended [11].⁠ Less invasive surgical procedures include needle aspiration of the vitreous fluid to reduce vitreal volume and possibly attempt to break up the anterior hyaloid [12, 13].⁠ More invasive procedures include vitrectomy, iridectomy, zonulectomy, capsulectomy, synechialysis, AC reformation, hyaloidectomy, and various combinations of those mentioned [2].

## 2. Case Report

In early 2020, an 84-year-old African American male with history of primary open angle glaucoma (POAG) was referred to the practice for evaluation and management of uncontrolled IOP. Prior to this exam, he had had standard phacoemulsification cataract extraction in both eyes, 180° selective laser trabeculoplasty (SLT) twice in his left eye (2014 and 2016), and a fornix-based trabeculectomy in his left eye (2014) all with our practice. He had also had blebitis in the left eye (2015) which was treated with topical antibiotics. The patient had systemic history of hypertension which was being treated with medication. The patient was currently taking latanoprost 0.005% qhs OU and timolol 0.5% BID OU and reported good compliance.

Visual acuities (uncorrected) were 20/20-2 OD and 20/25-3 OS. There was a grade 2+ relative afferent pupillary defect (RAPD) in the left eye. The bleb in his left eye was superior, low, cystic, and avascular, showed mild pigment speckling, and was Seidel negative. There was a patent iridectomy OS. Anterior chambers were deep and quiet in both eyes. Clear posterior chamber intraocular lenses (PC-IOL) in both eyes were intact and well positioned in the bag with posterior capsule intact. The Goldmann applanation tonometry (GAT) measured IOPs of 21 and 32 mmHg in the right and left eyes, respectively. Gonioscopy revealed that angles were bilaterally open to grades 3-4 (Shaffer-Kanski). Prior axial length and keratometry readings were 22.65 mm and 22.96 mm and 44.87/45.00@180 and 44.35/44.94@175 in the right and left eyes, respectively. Dilated fundus exam (DFE) showed glaucomatous cupping OD (cup-to-disc ratio 0.60V 0.60H) and severe glaucomatous cupping OS (0.95V 0.95H) with no Drance hemorrhages present in either eye. All other anterior segment and posterior segment findings were otherwise unremarkable.

**Figure 1:**
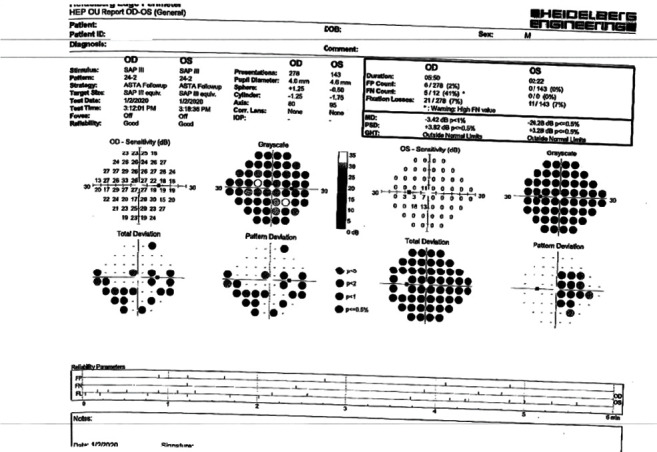
Heidelberg Edge Perimeter 24-2 of right eye and left eye.

**Figure 2:**
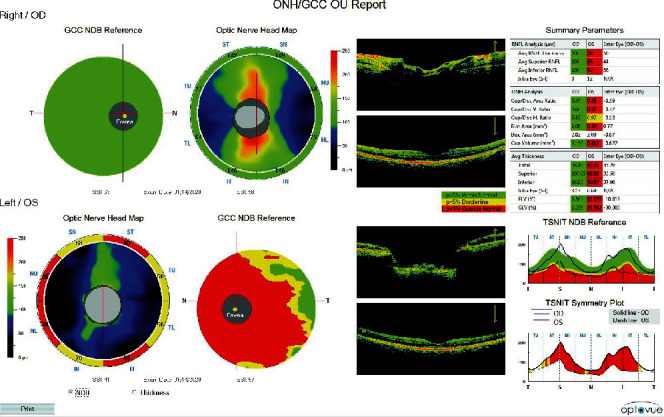
Combined ocular coherence tomography of the retinal nerve fiber layer and ganglion cell complex of the right eye (upper) and left eye (lower).

Recent Heidelberg Edge Perimeter (HEP) (Heidelberg Engineering Inc.; Franklin, MA) ASTA Follow-Up (Adaptive Staircase Thresholding Algorithm) 24-2 OD and 24-2 OS showed a new inferior arcuate defect OD and severe loss OS with only a small central island remaining (Figure 1). Optical coherence tomographies (Optovue RTVue; Fremont, CA) of the optic nerve head retinal nerve fiber layer and ganglion cell complex (OCT ONH RNFL and GCC) showed that OD RNFL and GCC were within normal limits, but OS RNFL and GCC were severely thinned (Figure 2). Given the right eye's history of elevated IOPs, glaucomatous cupping, visual field progression, and RNFL progression (“progression in the green”), IOP in the right eye was treated accordingly.

Based on these findings, we planned to perform SLT OD in office and to insert a XEN gel ab externo OS. The patient would continue latanoprost qhs OU and timolol 0.5% BID OU for now but discontinue glaucoma drops OS after XEN surgery.

The glaucoma specialist implanted the XEN gel stent ab externo in the superotemporal sector without complication. At the postop day 1 exam, OS visual acuity (VA) uncorrected was 20/25-3 and IOP was 10 mmHg without any drops. The anterior chamber was formed, and the XEN bleb was forming. The patient was instructed to begin ciprofloxacin 0.3% QID OS and prednisolone 1% q2h OS and continue his normal glaucoma drops OD only. Standard postop instructions and warnings were given instructing that he should call the office or our on-call system if he should notice any ocular pain or decrease in vision.

**Figure 3:**
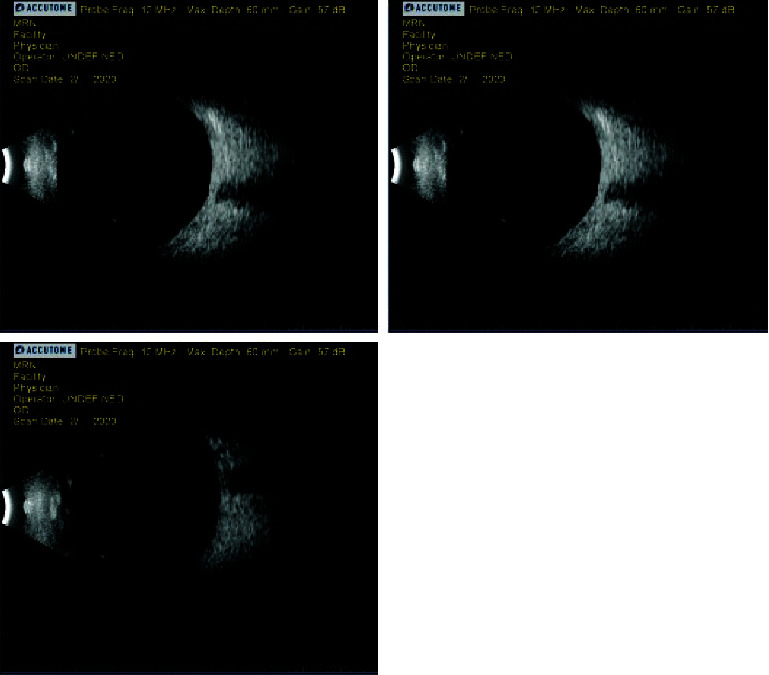
Ultrasound B-scan of the left eye on postop day 7. No choroidal detachment or hemorrhage.

The patient presented for his postop day 7 exam and reported severe pain and decreased vision in the left eye but was unclear concerning when both had started. Uncorrected visual acuity OS was light perception (LP) and did not improve with pinhole. The XEN bleb was low, diffuse, avascular, and slowly forming but was Seidel negative. The older trabeculectomy bleb had remained low and cystic, with mild pigment speckling, and was Seidel negative. The conjunctiva surrounding the XEN bleb was injected and had a subconjunctival hemorrhage. The cornea was mostly clear, and the anterior chamber (AC) was shallow centrally and peripherally but still formed. The lens-iris diaphragm was anteriorly displaced, though the iridectomy was patent and there was no iris bombé. Undilated view of the vitreous showed no hemorrhage. IOP OS was 65 mmHg. Ultrasound B-scan (Accutome 4Sight; Malvern, PA) confirmed no vitreous hemorrhage or choroidal detachment (Figure 3). Ultrasound biomicroscopy (UBM), unfortunately, could not be performed as our instrument was inoperable at that time. Based on these findings, ciliovitreal block was diagnosed.

The patient was administered two 250 mg tablets of acetazolamide, 6 fl oz of 45% isosorbide PO, 2 gtts Betimol 0.5% OS (Oak Pharmaceuticals Inc.; Lake Forest, IL), and 2 gtts cyclopentolate 1% OS. The patient was then sent to the ambulatory surgery center for immediate Nd-YAG (neodymium-doped yttrium aluminum garnet) laser posterior capsulotomy and anterior hyaloidotomy. On arrival, despite the cyclopentolate and glaucoma medications, IOP was still 65 mmHg and the AC was still shallow. YAG posterior capsulotomy and anterior hyaloidotomy were performed, but the AC did not deepen. As a result, the patient was prepped for surgery and the glaucoma specialist performed a pars plana core vitrectomy, anterior and posterior synechialysis, and AC reformation.

**Figure 4:**
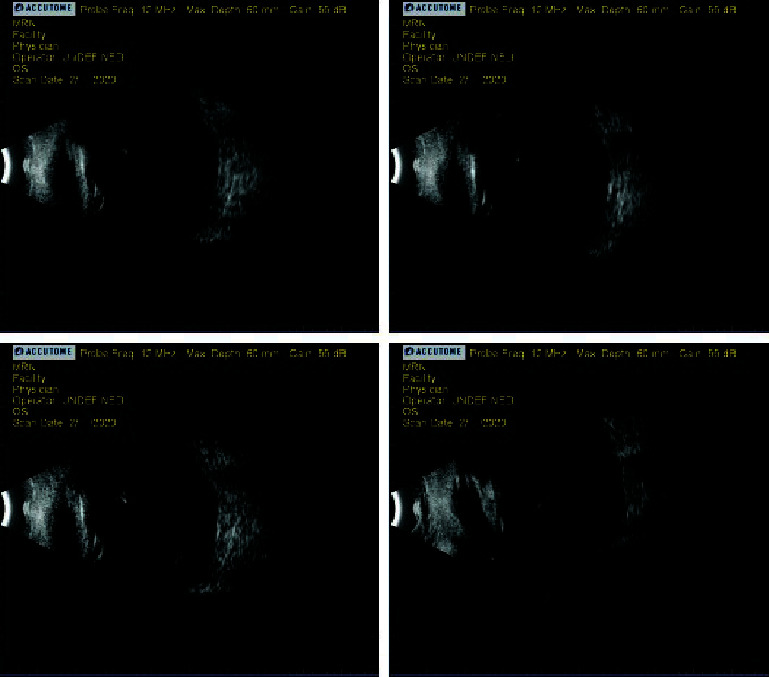
Ultrasound B-scan from postop day 1 (following vitrectomy). Mild vitreous haziness but no choroidals present.

At the postop exam the following day, the patient was using his usual glaucoma drops OD and prednisolone QID OS, ciprofloxacin QID OS, and cyclopentolate q2h OS (the patient likely had confused some of the instructions). He had also taken one Diamox Sequel (acetazolamide 500 mg extended release) (Teva Pharmaceuticals Industries Ltd.; Tel Aviv, Israel) that morning and was not experiencing significant adverse effects. Visual acuity OS was LP, and IOP via iCare (iCare IC100; iCare USA Inc.; Kansas City, KS) was 25 mmHg. The conjunctiva showed mild chemosis and injection, and the XEN bleb was still low but developing. There was dense corneal edema and diffuse pigment on the endothelium. The temporal corneal wound suture was intact. Anterior segment views were poor due to corneal haziness, but the AC appeared more formed. B-scan showed some vitreous haze, possibly suggestive of vitreous hemorrhage, but no frank choroidal detachment (Figure 4). Standard postop warnings and instructions were given. Medication instructions were clarified so he would use prednisolone 6-8×/day OS, ciprofloxacin QID OS, cyclopentolate BID OS, and Diamox Sequels (500 mg XR) BID PO. Given the likely continued systemic acetazolamide use during this recovery period, we discontinued his glaucoma medications in the right eye for the time being. The patient was also advised to increase his potassium intake to offset the potassium excretion caused by acetazolamide.

The following day 6, postop exam was similar. Visual acuity OS was LP, and IOP OS via GAT was 15 mmHg (on the correct medication regimen specified earlier). The XEN bleb was low but mildly vascular and developing. The cornea was still very hazy with dense pigment on the endothelium. The AC, however, was formed, and the iridectomy could be visualized as patent. Ciprofloxacin was discontinued, but all other medications were continued unchanged.

**Figure 5:**
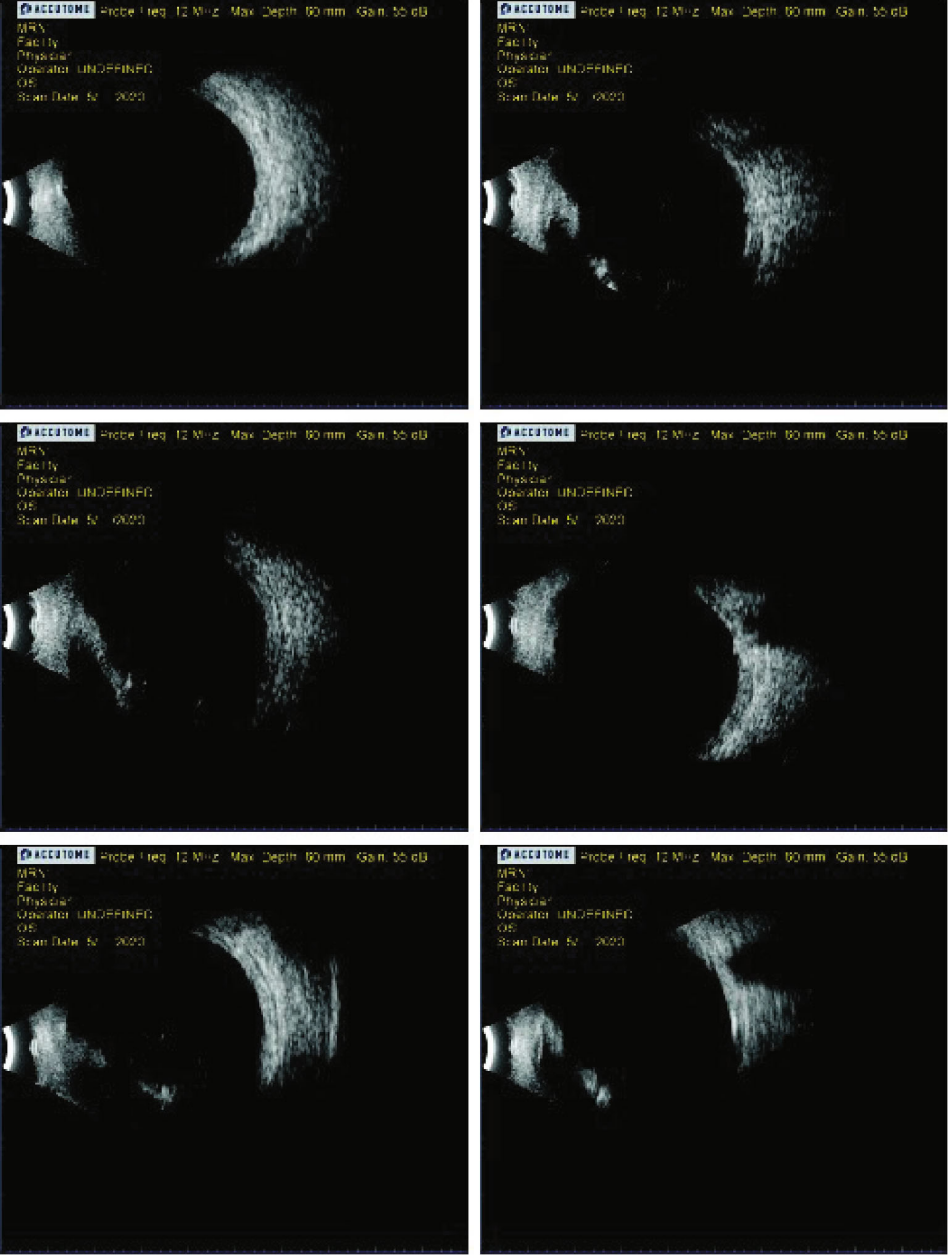
Ultrasound B-scan from postop day 90 (following vitrectomy). Mild vitreous haziness and visual artifacts present but no choroidals.

Multiple postop exams were conducted on days 15, 20, 35, and 90. At these exams, visual acuity OS was HM to LP, and IOP OS typically was in the midteens. Medications were tapered down very slowly with acetazolamide and cyclopentolate only being discontinued after the day 90 postop and the steroid continuing to be tapered past that. Corneal edema slowly improved, but the dense endothelial pigment clumps remained. On the day 90 postop, the XEN gel stent was situated close to but not in contact with the corneal endothelium. B-scan at that exam showed no choroidals but some haziness in the vitreous (Figure 5).

Surgical explantation of the XEN and referral to our cornea specialist for a DSAEK evaluation (Descemet's stripping automated endothelial keratoplasty) were considered. However, while vision was briefly HM for several postop exams, it remained LP after the day 90 postop.

Unfortunately, mild-moderate corneal edema OS did intermittently recur in the months following, possibly due to a combination of intermittent XEN gel contact with the endothelium and/or general corneal decompensation from multiple traumas. Prednisolone was continued at BID OS to prevent further corneal inflammation and preserve whatever XEN function still remained. At his most recent exam, though, IOP had risen to 21 and 32 mmHg in the right and left eyes, respectively. As a result, Lumigan (Allergan; Irvine, CA) was started qhs OD only. Prednisolone was decreased to QD OS only, and the patient was instructed to punctal occlude following prednisolone instillation to prevent potential crossover that could cause steroid response in the right eye. Focus shifted to lowering OD IOP to protect vision as best as possible and manage OS IOP for comfort.

## 3. Discussion

Ciliovitreal block is a form of secondary angle closure which typically occurs after surgery or exposure to certain medications (like pilocarpine [14]⁠), though it has been noted to happen in patients with no history of ocular surgery or trauma [15].⁠ Unlike the more common primary angle closure, ciliovitreal block can occur despite a patent LPI and the AC is shallow centrally and peripherally (while primary angle closure typically only shallows the peripheral AC). The lens-iris diaphragm is often displaced anteriorly, and the ciliary processes are usually anteriorly rotated [1].⁠ Additionally, unlike the more common hypotony-associated AC shallowing which can occur following filtering surgeries, ciliovitreal block is not associated with hypotony. IOP is usually elevated but can be normal if a functional trab or tube is present [2].⁠

Onset can be intraoperative but typically occurs within 1-2 months after surgery. Ciliovitreal block has been reported as occurring following phacoemulsification cataract surgery, glaucoma filtering surgery (including trabeculectomy and seton), pars plana vitrectomy (PPV), LPI, YAG capsulotomy, and retinal photocoagulation, among other procedures [1–3].⁠

The XEN 45 gel stent (Aquesys Inc.; Aliso Viejo, CA) used in this case is a relatively newer device made of porcine gelatin designed to be inserted into the AC with its distal end situated in the subconjunctival space to form a filtering bleb [16].⁠ The XEN was originally designed and FDA-approved to be inserted ab interno (from inside the AC), but our practice had not been satisfied with the outcomes using this method. As a result, our glaucoma specialist converted to inserting the XEN ab externo (from outside the eye) into the AC. Off-label ab externo implantation allowed greater control of the distal end's placement in the subconjunctival space which, in our cases, led to better IOP control. Previous cases have described ciliovitreal block following ab interno XEN placement, but this is the first, to our knowledge, case of ciliovitreal block occurring after ab externo XEN implantation [17–19].⁠

⁠We had decided to not wait and see if medication therapy would resolve his case and proceed directly with YAG capsulotomy/hyaloidotomy based on the high IOP and his already extensive glaucoma which, as mentioned earlier, had progressed to only leaving him a small central island of vision. We might have been more willing to treat with medications—only for several days had the IOP been lower or the right eye's glaucoma not as severe as it was, but his situation was too precarious. We had also had extensive discussions with the glaucoma specialist concerning the XEN gel stent's implantation and found no complications or anomalous events during the surgery which may have injured the ciliary body or otherwise precipitated the block or placed the patient at increased risk for developing ciliovitreal block.

There was some uncertainty as to the cause of the clumps of pigment on the corneal endothelium and what effect it had on the patient's vision. The anterior chamber may have completely flattened for a period of time between the surgery and the first postop exam, thus explaining the dense endothelial pigment. Alternatively, the surgical trauma may have liberated extensive amounts of pigment which deposited on the corneal endothelium.

We initially hypothesized that vision may have remained poor (HM to LP) due to the endothelial pigment but later concluded that it was likely due to irreversible glaucomatous damage to the optic nerve which had occurred due to the prolonged elevated IOP. As stated earlier, there was some uncertainty concerning how long the ocular pain had been present; for so, there may have been several days of extremely high IOP. There was already end-stage glaucomatous damage with severe central field loss present OS even before surgery, so damage to the remaining central field was the most likely culprit.

## 4. Conclusion

Although rare, ciliovitreal block should be considered as a differential in post-XEN gel stent patients who present with a shallow or flat anterior chamber. Prompt diagnosis and treatment can help prevent pain and vision loss from elevated IOP.

## Data Availability

The clinical data used to support the findings of this study are included within the article.
